# Superior X-ray Radiation Shielding Effectiveness of Biocompatible Polyaniline Reinforced with Hybrid Graphene Oxide-Iron Tungsten Nitride Flakes

**DOI:** 10.3390/polym12061407

**Published:** 2020-06-23

**Authors:** Seyyed Alireza Hashemi, Seyyed Mojtaba Mousavi, Reza Faghihi, Mohammad Arjmand, Mansour Rahsepar, Sonia Bahrani, Seeram Ramakrishna, Chin Wei Lai

**Affiliations:** 1 Department of Mechanical Engineering, Center for Nanofibers and Nanotechnology, National University of Singapore, Singapore 119077, Singapore; seeram@nus.edu.sg; 2 Department of Medical Nanotechnology, School of Advanced Medical Sciences and Technologies, Shiraz University of Medical Sciences, Shiraz 71348-14336, Iran; kmepo.smm@gmail.com (S.M.M.); s.bahrani22@gmail.com (S.B.); 3 Department of Chemical Engineering, National Taiwan University of Science and Technology, Taipei 10607, Taiwan; 4 Nuclear Engineering Department, Shiraz University, Shiraz 71936-16548, Iran; faghihir@shirazu.ac.ir; 5 Radiation Research Center, Shiraz University, Shiraz 71936-16548, Iran; 6 School of Engineering, University of British Columbia, Kelowna, BC V1V 1V7, Canada; mohammad.arjmand@ubc.ca; 7 Department of Materials Science and Engineering, School of Engineering, Shiraz University, Zand Boulevard, Shiraz 71348-51154, Iran; mansour.rahsepar@gmail.com; 8 Nanotechnology & Catalysis Research Center, University of Malaya, Kuala Lumpur 50603, Malaysia; cwlai@um.edu.my

**Keywords:** X-ray attenuation, biocompatible, polyaniline, graphene oxide, anti-bacterial/anti-fungal

## Abstract

X-ray radiation is a harmful carcinogenic electromagnetic source that can adversely affect the health of living species and deteriorate the DNA of cells, thus it’s vital to protect vulnerable sources from them. To address this flaw, the conductive polymeric structure of polyaniline (PANi) was reinforced with diverse filler loadings (i.e., 25 wt % and 50 wt %) of hybrid graphene oxide-iron tungsten nitride (ITN) flakes toward attenuation of X-ray beams and inhabitation of microorganisms’ growth. Primary characterizations confirmed the successful decoration of graphene oxide (GO) with interconnected and highly dense structure of iron tungsten nitride with a density of about 24.21 g·cm^−3^ and reinforcement of PANi with GO-ITN. Additionally, the outcome of evaluations showed the superior performance of developed shields, where a shield with 1.2 mm thickness containing 50 wt % GO-ITN showed 131.73% increase in the electrical conductivity (compared with neat PANi) along with 78.07%, 57.12%, and 44.99% decrease in the amplitude of the total irradiated X-ray waves at 30, 40, and 60 kVp tube voltages, respectively, compared with control X-ray dosage. More importantly, the developed shields not only showed non-toxic nature and improved the viability of cells, but also completely removed the selected microorganisms at a concentration of 1000 µg·mL^−1^.

## 1. Introduction

Development of nuclear-based technologies and usage of X-ray waves as an ionized radiation source for diverse applications—e.g., radiation therapy, medical applications, imaging, space technology, and agricultural applications—have improved the interaction of living species with these kinds of harmful electromagnetic sources [1,2,3,4,5,6,7,8]. Receiving high dosage of ionizing radiation can be lethal to living cells by deteriorating/damaging the DNA of their cells. Besides, despite the fact that cells can repair themselves after being exposed to low dosage of ionizing radiation, but in some cases, cells do not fully recover, leading to cancer and thus death of the exposed person [9]. Therefore, development of multifunctional radiation shields for protecting vulnerable sources from ionizing radiation sources is imperative.

Employed radiation sources during medical scanning via imaging devices such as CBCT can easily affect sensitive organs (e.g., thyroid) and in some cases cause mutagenic, carcinogenic, and chronic health effects on diverse parts of the body [10,11,12]. Many attempts were taken to develop practical shields to protect people and vulnerable sources from ionizing radiation. In this case, polymeric-based composites reinforced with high-density additives such as lead and tungsten found to be ideal candidates [13,14,15]. In a series of works by Azman et al. [13,14], they developed shields against ionizing radiation (i.e., X-ray radiation) based on reinforced epoxy composite with tungsten trioxide (WO_3_), where the developed shields were examined using mammography and radiation radiography units. Achieved results showed that the particle size has a significant effect on the X-ray attenuation rate at tube voltage under 35 kVp, while at tube voltage more than 40 kVp up to 120 kVp, the effect of particle size is negligible. In another attempt, Hashemi et al. [15] used the graphene oxide (GO) as a carrier for loading lead oxide particles toward development of a 2D planar X-ray shield, where the developed flakes were used to fabricate epoxy-based composites at diverse filler loadings (i.e., 5 and 10 wt %) and thicknesses (i.e., 4 and 6 mm). The outcome of their research showed that composites containing 10 wt % GO-Pb_3_O_4_ with 6 mm thickness exhibited absorption rates equivalent to 4.06, 4.83, and 3.91 mm Al at 40, 60, and 80 kVp energies. This is equivalent to 124.3%, 124.6%, and 103.6% improvement in the X-ray absorption rate compared with neat epoxy matrix, respectively.

Additionally, GO is a potential 2D material that can be integrated with high-density materials such as Pb through their active functional groups and leads to their homogeneous distribution within diverse polymeric matrices. This is vital for fabricating a practical and efficient lightweight composite shield for absorption of X-ray radiation. In this case, reinforcement of epoxy composite with GO-Pb_3_O_4_ not only can lead to uniform dispersion of Pb within a polymeric matrix, but also improve the corrosion resistance, mechanical properties and thermal properties of the final composition. It is reported that a composite containing 10 wt % GO-Pb_3_O_4_ can fully resist against highly corrosive solvents (e.g., xylene-o and xylene-m) and improve the hardness, contact angle and glass transition temperature (T_g_) about 0.9 kgf·mm^−2^, 27.8°, and 53.23 °C, respectively [15,16].

Polyaniline (PANi) is a potential polymeric matrix that can be easily coupled with diverse additives, be loaded with a high amount of fillers, and used as a potential X-ray shield. In this context, Hosseini et al. [17] reinforced PANi with diverse weight percentages of lead (10–40 wt %) at different thicknesses (0.5–6 mm) toward X-ray radiation shielding. Their obtained results showed that further increase in the weight percentages of lead and thickness of the final composition can considerably improve the X-ray attenuation rate at tube voltages ranging from 14 to 30 kVp.

One of the main disadvantages of common radiation shields filled with high-density elements (e.g., Pb and W) is their high weight and severe toxicity, which makes them hazardous for life of living species. For instance, the US Agency for Toxic Substances and Disease Registry (ATSDR) Substance Priority List recognized lead as the second most toxic element for humans that can impair proper function of enzymes and adversely affect the metabolism of calcium within the body [18]. Likewise, Pb is also known as a neurotoxicant element that can deteriorate and disturb the proper function of the brain [19]. To address these flaws and obstacles, first, we developed hybrid 2D flakes consisting of decorated GO with interconnected iron tungsten nitride (GO-ITN) patterns. Then, polyaniline (PANi) was filled with diverse filler loadings (i.e., 25 and 50 wt %) of the as developed hybrid flakes (with respect to the aniline monomer) toward development of biocompatible and non-toxic X-ray radiation shield. The developed shields can also act as an anti-bacterial/anti-fungal agent against diverse kinds of microorganisms and minimize nosocomial infections.

## 2. Experimental

### 2.1. Materials

For the synthesis of developed nanomaterials and nanostructures, all of required materials—including ultrapure graphite powder, potassium permanganate (KMnO_4_), sulfuric acid (H_2_SO_4_), ortho-phosphoric acid (H_3_PO_4_), hydrogen peroxide (H_2_O_2_), hydrochloric acid (HCl), ethanol (C_2_H_5_OH), acetone (C_3_H_6_O), aniline monomer, ammonium persulfate ((NH_4_)_2_S_2_O_8_), ammonia (NH_3_), iron (III) chloride hexahydrate (FeCl_3_·6H_2_O), iron (II) sulfate heptahydrate (FeSO_4_·7H_2_O), and tungstic acid (H_2_WO_4_)—were supplied by Merck & Co., Inc (Darmstadt, Germany). All of the reagents were used as received except aniline monomer that was vacuum distilled before use.

### 2.2. Methods

#### 2.2.1. Synthesis of Graphene Oxide

To synthesize GO out of graphite flakes, first, 720 mL H_2_SO_4_ and 80 mL H_3_PO_4_ were added together and stirred (500 rpm) for 10 min at room temperature (RT) followed by addition of 6 g ultrapure graphite powder and stirred for further 30 min at RT. Then, the temperature of the suspension was reduced to less than ~5 °C using an ice bath, followed by slow addition of 36 g KMnO_4_ over 30 min. In the next step, the temperature of the suspension smoothly increased to 50 °C and stirred (500 rpm) for 48 h. Next, the temperature of the suspension decreased to RT and after that, a solution containing H_2_O/H_2_O_2_ was added to the resulting suspension. In this step, the temperature of the suspension increased to 95 °C, where the suspension was kept at this temperature for 15 min and then cooled to RT using an ice bath. The resulting suspension was then ultrasonicated for 1 h at 600 W, kept stationary for 24 h, and the stable upper solution was collected for further use. Afterward, the resulting materials were filtrated using 0.22 µm PTFE filter paper and washed with deionized (DI) water, 30 vol% HCl, and DI water, respectively. Finally, the resulting powder was placed in an oven at 100 °C for 2 h and then kept in desiccator for further use. Figure S1 illustrates a view of GO’s production steps.

#### 2.2.2. Synthesis of Hybrid Graphene Oxide-Iron Tungsten Nitride Nanoflake

In order to synthesize decorated GO with iron tungsten nitride, a multi-stage manufacturing procedure was designed and employed. For this matter, first, 3.688 g well-exfoliated GO powder was added to 100 mL ethanol, and then ultrasonicated for 30 min (200 W, 300 W, and 400 W for 10 min, respectively). In the next step, 10 g tungstic acid (H_2_WO_4_) was added to the suspension and ultrasonicated for 10 min at 300 W. Next, 320 mL DI water was added to the suspension and ultrasonicated for 20 min at 400 W. The resulting suspension was then poured into a flat-bottom balloon and stirred (500 rpm) for 1 h at 80 °C. Afterward, 3.89 g FeCl_3_·6H_2_O and 4.55 g FeSO_4_·7H_2_O were poured into the resulting suspension and stirred (500 rpm) for 1 h at 80 °C, followed by addition of 50 mL NH_3_ to the suspension. The resulting mixture was thence stirred (500 rpm) for 48 h at 80 °C under reflux. The final powder was filtrated and washed with DI water, until the resulting fine powder was neutralized. Then, the filtrated materials were placed within an oven and heated up at 100 °C for 2 h. As control, the Fe_3_O_4_ nanoparticles were also synthesized via a simple procedure according to our previous studies, where the related characterizations also exist [20,21,22,23].

#### 2.2.3. Fabrication Procedure of Reinforced Polyaniline with Graphene Oxide-Iron Tungsten Nitride Nanoflakes

For fabrication of PANi, first, 3.72 g highly purified vacuum distilled aniline monomer and also 9.1 g ammonium persulfate (APS) were separately added to 50 mL 1 M HCl followed by stirring (500 rpm) for 30 min at RT till gaining a homogenous suspension. Next, the temperature of the aniline monomer/1 M HCl solution was reduced to less than 5 °C using an ice bath followed by dropwise addition of APS/ 1 M HCl suspension to the monomer mixture. The resulting mixture was thence stirred (500 rpm) for 24 h at the same temperature till full polymerization of the aniline monomer. In the next stage, the resulting blackish green polyaniline emeraldine salt was filtrated and washed several times with 0.2 M HCl, acetone and DI water, respectively. Finally, the developed polyaniline powder was placed in a vacuum oven at 60 °C for 12 h.

For production of hybrid Polyaniline-GO-iron tungsten nitride nanostructures, 25 wt % (PANi-GFW 25%) and/or 50 wt % (PANi-GFW 50%) GO-ITN hybrid nanoflakes concerning the aniline monomer were added to the primary 1 M HCl solution and ultrasonicated for 30 min at 600 W. Then, the aniline monomer was added to the resulting suspension and the rest of the procedure was performed according to the polyaniline’s production procedure.

### 2.3. Characterization

To examine the developed nanomaterials and nanostructures, diverse analyses were used. In this matter, to confirm the successful synthesis/exfoliation of GO nanoflakes, X-ray diffraction (XRD) (Panalytical model X’Pert Pro, Almelo, Netherlands), micro Raman spectroscopy (Nicolet Almega thermo-dispersive Raman spectrometer, Kyoto, Japan), X-ray photoelectron spectroscopy (XPS) (Thermo ScientificTM K-Alpha XPS spectrometer, Waltham, MA, USA), transmission electron microscopy (TEM) (Jeol ARM 200, Akishima, Japan), and Fourier-transform infrared (FTIR) spectroscopy (Bruker model Tensor II, Billerica, MA, USA) were used. Moreover, as developed GO-ITN hybrid flakes were characterized via FTIR, field emission scanning electron microscope (FESEM) (Tescan model Mira III, Brno, Czech Republic) and energy dispersive spectroscopy (EDAX) (Tescan model S Max detector Mira III, Brno, Czech Republic). For evaluation of the reinforced PANi’s nanostructures, FTIR, XRD, FESEM, vibrating-sample magnetometer (VSM) (Lakeshore Cryotronics, Inc., model 7300, Oxford, UK) and Thermogravimetric analysis (TGA) (TG 209 F3 Netzsch, Selb, Germany) were used.

### 2.4. X-ray Shielding Evaluation of Developed Nanostructures

The X-ray attenuation examination was performed on reinforced PANi’s composites with 25 or 50 wt % GO-ITN hybrid flakes with respect to the primary aniline monomer’s weight, which was produced through molding the specimens via hydraulic press at 10 MPa pressure for several minutes. The related specimens were fabricated with 2 cm diameter and at diverse thicknesses. The specifications of developed samples are tabulated in the Table S1.

Afterward, the developed specimens were placed between the probe of the mammography unit (Solidose RT100 B, Mölndal, Sweden) and X-ray detector with 90 cm distance from the probe of the mammography unit where X-ray beams were emitted to samples through a highly concentrated focal spot. In this matter, diverse X-ray dosages were emitted to samples via variating the tube voltage, where at 30, 40, and 60 kVp tube voltages the current/time was set on 1 mA/1 s, 10 mA/1 s, and 10 mA/0.5 s, respectively. Moreover, for higher tube voltages, i.e., 40 and 60 kVp, the radiology probe was utilized and +1 and +2 mm Al was used for filtration of X-ray beams, respectively.

### 2.5. Anti-Bacterial and Anti-Fungal Evaluation of Developed Shields

The anti-bacteria and anti-fungal performance of developed shields was examined via minimum inhibitory concentration (MIC), minimum bactericidal concentration (MBC), and minimum fungal concentration (MFC) tests. In this matter, two diverse gram positive (i.e., *Staphyloccus aureus* and *Enterococcus faecalis*) and gram negative bacteria (i.e., *Escherichia coli* (*E. coli*) and *Pseudomonas aeruginosa*) were used for anti-bacterial evaluations, and a yeast (i.e., *Candida Albicans*) was selected to check the anti-fungal performance of developed samples.

The related evaluations were performed according to the guideline of the Clinical and Laboratory Standard Institute (CLSI), in which a dilution (from 1000–7.8 µL) guideline was proposed for checking MIC, MBC/MFC of developed agents [24,25]. In this regard, the MIC and MBC/MFC of specimens were checked via the lowest concentration of antimicrobial/anti-fungal that the developed agent inhibited the growth of a microorganism of about 90% after 24 h incubation in comparison with the negative control. For MIC, MBC/MFC examinations, firstly, serial dilutions of developed shields’ powder were prepared and poured in a 96-well microplate with 96 µL Mueller–Hinton Broth (MHB) medium. The prepared microbial/yeast strains were cultured within the MHB to reach the turbidity of about 5 × 10^8^ CFU/mL (in OD_600_). In the next step, the related bacterial/yeast suspensions were produced for inoculation through diluting them to yield 5 × 10^6^ CFU/mL. After that, 10 µL of each as prepared suspension of bacteria/yeast was inoculated into considered microplates; after elapse of 24 h incubation at 37 °C, the related optical density of each well was calculated using a micro-plate reader (BioTek, Power WaveXS2, Winooski, VT, USA) at wavelength of 600 nm. In this work, brain heart infusion (BHI) was considered as the negative control, and combination of BHI and culture media were selected as positive control. Additionally, the achieved data from the anti-bacterial/anti-fungal evaluation were examined using SPSS IBM software.

### 2.6. Toxicity Evaluation of Developed Shields

In the current research, the MCF-7 cell line was used for in vitro cytotoxicity assay of the developed shields. In this matter, first, the BRMI that was supplemented with 10% fetal bovine serum (FBS) and 5% penicillin-streptomycin as the medium for cell culture. Thence, cells were cultivated within a humid atmosphere with 5% CO_2_ gas injection at temperature of about 37 °C. Next, the cytotoxicity of PANi and reinforced PANi with diverse filler loadings (i.e., 25 and 50 wt %) of GO-ITN was examined against as cultured cells (here MCF-7) using the MTT colorimetric assay. For this regard, cultured cells were injected into 96-well plates with about 2000 cells in each well. In the first step, the cultured MCF-7 cells were incubated for 24 h and then treated with diverse concentrations of developed shields’ powder. Next, cells were incubated for 24 h at 37 °C under 5% CO_2_ followed via replacement with 30 µL MTT:PBS solution (4 mg·mL^−1^). Thereafter, the as-prepared plates were incubated for 3 h at 37 °C under 5% CO_2_, followed by addition of 100 µL DMSO to each well for dissolving insoluble Formazan crystals. In the final step, the absorbance of each respective well was determined via microplate reader (BioTek, Power WaveXS2) at wavelength of 600 nm. Then, the viability of cells was determined using the formula

(1) $$\mathrm{Viability}\;\left(\%\right)=\frac{A_{t}-A_{b}}{A_{c}-A_{b}}\times 100$$

where A_t_, A_b_, and A_c_ are attributed to the absorbance in each experiment well, absorbance in the witness well, and absorbance in control well, respectively. Herein, the achieved results from the MTT assay were examined using SPSS IBM software.

## 3. Results and Discussion

### 3.1. Characterization of Developed Nanomaterials and Nanostructures

**Figure 1:**
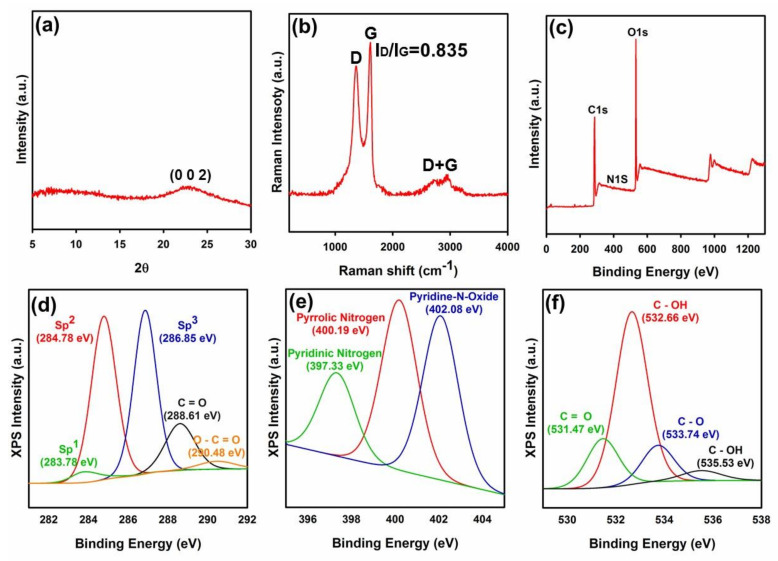
(**a**) XRD result of the developed GO, (**b**) micro Raman spectroscopy of GO, (**c**) XPS elemental analysis of GO; De-convolution of GO XPS peaks (**d**) C1s, (**e**) N1s, and (**f**) O1s into diverse segments.

In this section, developed nanomaterials and nanostructures were evaluated through diverse analyses to confirm their successful synthesis/fabrication. In this matter, the outcome of performed evaluations showed that the developed GO nanoflakes were synthesized in good order and high quality. In Figure 1a, XRD diffractogram of fabricated GO flakes can be seen. As can be seen in this Figure, well-resolved diffraction peak of the (002) plane was observed at 2θ of 23.08 which shows the successful exfoliations of GO from bulk graphite flake. Raman spectrum of the fabricated GO is illustrated in Figure 1b. Raman spectrum showed D, G, and D + G band peaks at 1374.55, 1606.69, and 2942.91 cm^−1^, respectively, while the ratio of I_D_/I_G_ was 0.83. The D band peak arises from the A_1g_ symmetry and zone boundary phonons and is ascribed to structural defects or the breakdown of the translational symmetry in the graphite lattice. On the other hand, the G band is the first-order scattering, corresponding to the E_2g_ symmetry and Brillouin zone of crystalline sp^2^ lattices of graphite [26,27,28,29]. The D + G band peak is engaged with a combination of D and G modes and signifies the disorders in the graphitic structure. Hence, the lower the I_D_/I_G_ ratio, the lower the structural defects [27,28,29]. A comparison between I_D_/I_G_ ratio of the current method and previous methods is compiled in Table S2. This comparison reveals that fabricated GO contains a low density of structural defects. Indeed, the current method presents a I_D_/I_G_ ratio smaller than reduced GO (rGO), validating graphene oxide with minimized defects in the basal plane.

XPS results for the fabricated GO are displayed in Figure 1c–f. Primary evaluation by the XPS analysis showed that the synthesized GO comprised from 68.36, 31.00, and 0.65 atomic % carbon, oxygen, and nitrogen (Figure 1c and Table S3). De-convolution of peak C1s (Table S4) showed that fabricated GO held 3.33, 41.62, and 55.05 atomic % sp^1^ hybridization carbon, sp^2^ hybridization carbon, and oxidized carbon structure (i.e., sp^3^ hybridization carbon, C=O, and O–C=O), respectively. Moreover, de-convolution of peak N1s (Table S5) revealed that the synthesized GO consisted of 19.87, 40.79, and 39.35 atomic % pyridinic nitrogen, pyrrolic nitrogen, and either pyridine-N-oxide or graphitic nitrogen, respectively. While, de-convolution of peak O1s (Table S6) showed that the phenolic functional groups of GO consisted of 18.08, 63.83, 13.44, and 4.65 atomic % C=O, C–OH, C–O, and C–OH, respectively.

In Figure 1d, C1s peak of GO is de-convoluted into five chemically shifted segments with binding energies at 283.78, 284.78, 286.85, 288.61, and 290.48 eV. In this regard, segments at binding energies 283.78, 284.78, and 286.85 eV correspond to sp^1^ carbon hybridization, non-oxygenated carbon C–C/C–H or sp^2^ carbon hybridization (graphitic structure), and interaction of carbon atoms with epoxide (C–O) or hydroxyl (C–OH) functional groups (sp^3^ hybridization), respectively [26,30,31,32]. Besides, the segment with binding energy of 288.61 eV is attributed to the carbonyl group (C=O) [26], while the segment at the binding energy of 290.48 corresponds to the carboxylate functional group (O–C=O) [33,34].

Figure 1e shows the de-convolution of peak N1s into three diverse segments corresponding to pyridinic nitrogen (397.33 eV), pyrrolic nitrogen (400.19 eV), and pyridine-N-oxide (402.08 eV) [35]. Pyridinic nitrogen denotes nitrogen with a lone electron pair located either at the edge of the GO network or next to a vacancy, bonded to two carbon atoms [35,36]. Pyrrolic nitrogen is a type of nitrogen atom positioned within a five-membered ring, while pyridine-N-oxide is attributed to various oxidized nitrogen configurations. On the other hand, in some cases, it is reported that binding energies of about 402–403.5 eV are attributed to clustered N substitutions (i.e., graphitic nitrogen) [35].

The phenolic functional groups within the fabricated GO were evaluated by the de-convolution of singlet O1s into four diverse segments (Figure 1f). The segments are engaged with C=O double bond to aromatic carbon (531.47 eV), C–OH carbon single bond to the hydroxyl group (532.66 eV), C–O oxygen single bond to carbon (533.74 eV), and C–OH (535.53 eV) [32]. Besides, the chemical shift of these peaks to slightly smaller binding energies compared with other methods [26,33] could be related to the more substantial electronic charge on oxygen atoms and better exfoliation of the fabricated GO [26]. These results are in good accord with the obtained data from XRD and micro Raman spectroscopy analyses.

Obtained results showed that fabricated GO contained various hydrophilic functional groups along with an improved graphitic carbonaceous structure. In accord with the previous analyses, the outcome of the XPS results showed a significant value in the intensity of sp^2^ hybridization (284.78 eV). Table S7 compares the obtained XPS and XRD results via Hummers, modified Hummers, and improved Hummers methods with the current method. As presented, fabricated GO presents lower layer-to-layer distance and enhanced graphitic carbon structure (i.e., C=C bond or sp^2^ carbon hybridization) compared with the other procedures. These data vividly confirm the successful synthesis of well-exfoliated GO with fine graphitic carbonaceous structure.

**Figure 2:**
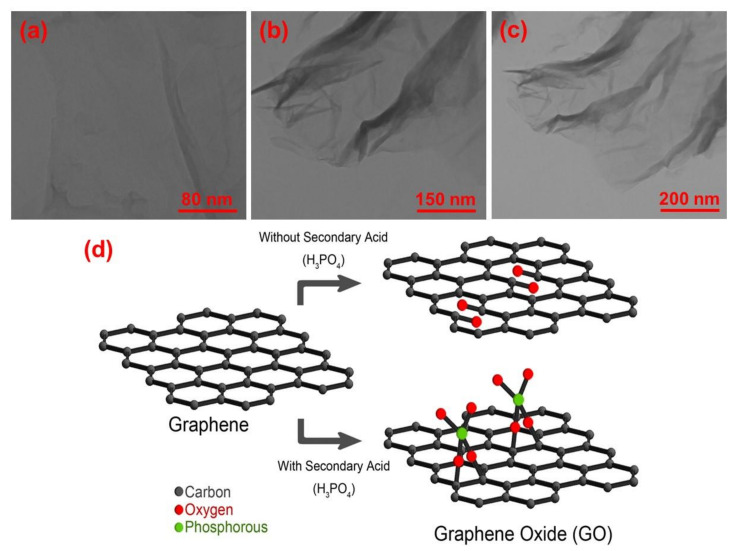
(**a**–**c**) A view of GO’s TEM images at diverse scales (i.e., 80, 150, and 200 nm) and (**d**) performance of secondary acid (H_3_PO_4_) in minimizing the overall amount of defects throughout the graphitic carbonaceous structure.

Additionally, in accord with previous analyses, the outcome of TEM analysis also confirmed the successful exfoliation of GO flakes which can be seen in Figure 2a–c. As can be seen in this Figure, the developed GO flakes were well-exfoliated and presenting ideal active surface area for modification with external materials. Furthermore, extreme oxidation can profoundly affect the graphitic carbonaceous structure and create holes and irreversible changes and defects within the basal plane of graphene, all of which cannot be repaired even by chemical or thermal reduction process [33]. Moreover, the presence of these defects throughout the graphitic carbonaceous structure along with oxygen-based functional groups can deteriorate the electrical conductance of graphene sheets due to widening of the electronic bandgap in the graphitic structure [37]. To cope with the aforementioned major problems, H_3_PO_4_ as a secondary acid was proved to be a very effective in minimizing the overall amount of defects and holes throughout the graphitic carbonaceous structure (Figure 2d).

During the chemical process leading to the exfoliation of graphite and formation of GO, permanganate ions attack C=C double bonds, resulting in the creation of vicinal diols. In absence of H_3_PO_4_, permanganate ions transform these vicinal diols into the C=O carbonyl groups, thereby creating voids and defects within the GO structure [38]. In the presence of H_3_PO_4_, phosphorous attaches to the vicinal diols and prevents the creation of carbonyl groups, thereby avoiding the creation of voids and defects within the GO structure [39]. The successful defect preventing role of H_3_PO_4_ was proved via micro Raman analysis, where the value of I_D_/I_G_ was determined to be 0.83 (see Table S2 and Figure 1b).

**Figure 3:**
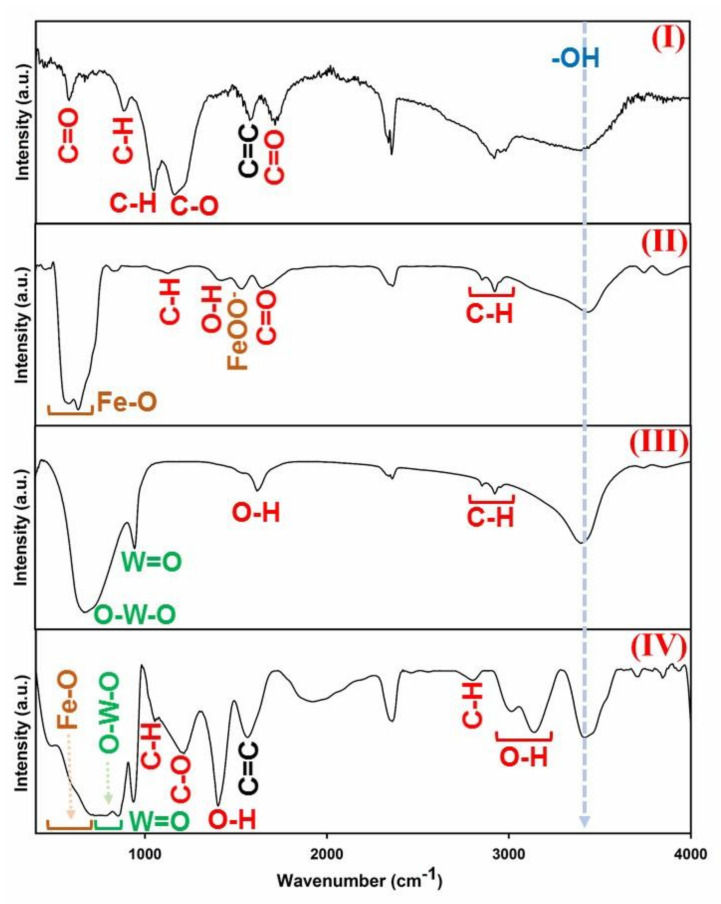
FTIR spectrum of (I) GO, (II) Fe_3_O_4_, (III) H_2_WO_4_, and (IV) GO-ITN hybrid flakes.

Developed GO flakes were used to fabricate hybrid GO-ITN sheets. To validate the successful synthesis of this hybrid structure, first of all used chemical compounds and the resulting hybrid flakes of GO-ITN were examined via FTIR analysis. In Figure 3, a view of GO, Fe_3_O_4_, H_2_WO_4_, and GO-ITN FTIR spectrums can be seen. As can be seen in Figure 3 part (I), the developed GO is synthesized with common active functional groups where each peak correspond to the C=O in amides (582 cm^−1^), C–H sp^2^ (882 cm^−1^), vibration of a p-disubstituted phenyl group ($v_{C-H}$ in-plane bending) (1049 cm^−1^), alkoxy C–O (1166 cm^−1^), bending of C=C from unoxidized sp^2^ double bond carbon atoms (1578 cm^−1^), stretching vibration of sp^3^ hybridization C=O bond (1716 cm^−1^) and hydroxyl functional groups (–OH) (3399 cm^−1^) [26,33,40,41,42,43,44].

Moreover, Fe_3_O_4_ nanoparticles were also synthesized in good order where the presented peaks in Figure 3 part (II) correspond to stretching vibration mode of Fe–O (584–635 cm^−1^), stretching vibration of in-plane C–H (1127 cm^−1^), vibration of O–H bond (1422 cm^−1^), FeOO^−^ (1534 cm^−1^), C=O stretching vibration (1649 cm^−1^), C-H stretching vibration related to sp^3^ stretching of hexyl aliphatic side (2855–2924 cm^−1^) and hydroxyl functional groups (–OH) (3434 cm^−1^). What is more, in Figure 3 part (III), the FTIR spectrum of H_2_WO_4_ can be seen. Typically, strong and broad absorption in the H_2_WO_4′_s FTIR spectrum within the range of 500–900 cm^−1^ is attributed to the O–W–O stretching mode, thus the appeared peak at 668 cm^−1^ corresponds to the fingerprint of O–W–O [45]. Additionally, other peaks within the H_2_WO_4′_s spectrum correspond to the W=O groups (954 cm^−1^), O–H bending vibrations (1617 cm^−1^), C–H stretching vibration (2854–2957 cm^−1^), and hydroxyl functional groups (–OH) (3393 cm^−1^) [45,46,47].

In Figure 3 part (IV), the FTIR spectrum of complex hybrid structure of GO-ITN can be seen. In this matter, broad and strong peak between 513–908 cm^−1^ contains mixed phases of Fe–O and O–W–O stretching vibrations that their fingerprints appear within ranges 530–630 cm^−1^ and 500–900 cm^−1^, respectively [20,47]. This strong peak confirms that both Fe_3_O_4_ and H_2_WO_4_ were coupled together and formed a doped structure and thence bonded with functional groups of GO via common active functional groups between them such as hydroxyl functional groups through covalent bonding. Additionally, peaks at wavelengths 936, 1054, and 1213 cm^−1^ are attributed to W=O, $v_{C-H}$ in plane bending and C–O functional groups, respectively. Moreover, other appeared peaks are correspond to the vibration of O–H bond due to the presence of surface-absorbed particles (1401 cm^−1^) [48], C=C sp^2^ carbon atoms (main finger print of graphene oxide) (1562 cm^−1^), C–H stretching vibration (2802 cm^−1^), carboxylic acid O–H functional groups (3104–3139 cm^−1^) and hydroxyl functional groups (–OH). These obtained data from FTIR analysis clearly confirmed the successful synthesis of GO-ITN hybrid structure and integration of magnetite nanoparticles with tungsten throughout the GO surface.

**Figure 4:**
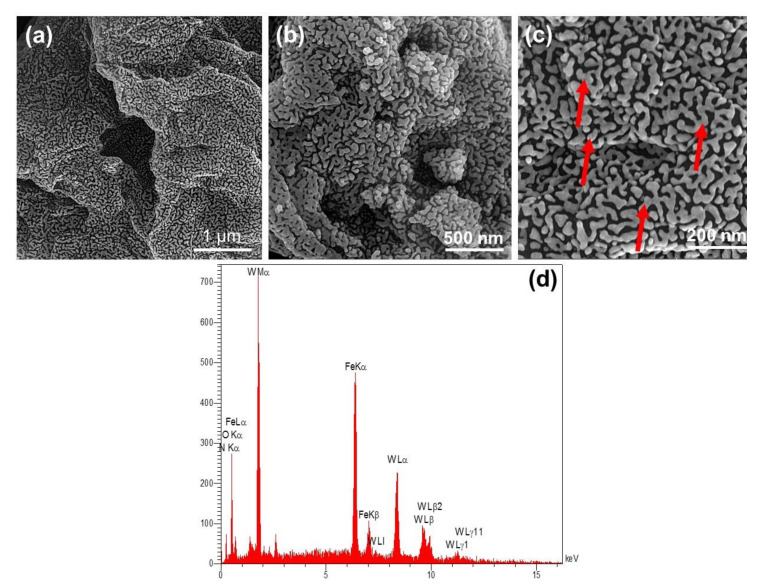
(**a**–**c**) FESEM images of hybrid GO-ITN at diverse scales (red arrows showing the interconnected iron tungsten nitride’s structure on the surface of GO) along with (**d**) related EDAX analysis of this hybrid platform.

In addition, In Figure 4a–c, FESEM images of hybrid GO-ITN can be seen at diverse scales (i.e., 1 µm, 500 nm, and 200 nm). As shown, the developed structure consisted of a planar structure decorated with randomly distributed interconnected iron tungsten nitride’s structure that covered the whole surface area of the GO and can create a strong barrier against incident X-ray waves. In Figure 4d, the EDAX analysis of GO-ITN can be seen. As shown, this hybrid planar structure mainly consists of 8.02, 1.87, 11.87, 13.39, and 64.85 weight percentage of carbon, nitrogen, oxygen, iron, and tungsten, respectively, where the hybrid platform mainly consisted of iron and tungsten; more details of EDAX analysis can be seen within Table S8. These outcomes are in well-accord with previously obtained data and confirm the successful synthesis of hybrid GO-ITN flakes.

**Figure 5:**
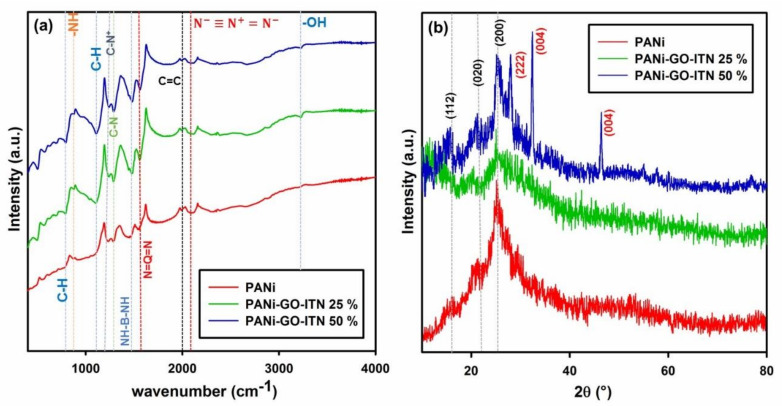
(**a**) FTIR and (**b**) XRD spectrums of PANi, PANi-GO-ITN 25%, and PANi-GO-ITN 50%.

Furthermore, the FTIR spectrum of PANi, PANi-GO-ITN 25%, and PANI- GO-ITN 50% can be seen in Figure 5a. As shown in this Figure, PANi and its reinforced composites showed common peaks of PANi with some variations due to the interaction of GO-ITN with the polymeric structure of PANi. In this case, peak in the region between 790–794 cm^−1^ is belong to the out of plane vibration of C–H functional group within the benzene ring of PANi [49], while peaks in the region between 873–881 cm^−1^ are correspond to the -NH vibrational modes. What is more, appeared peaks within the region between 1108–1125 cm^−1^ are attributed to the vibration of C-H related to the N=quinoid ring (Q)=N along with B-N^+^H-B bonds which are related to the charge carriers in the polymeric chains of PANi due to the protonation process. These peaks are significantly more intense and broader for PANi- GO-ITN 25% and PANi- GO-ITN 50% due to the existence of higher amount of C-H functional groups within the GO-ITN’s structure and higher level of hydrogen bonding between PANi and as developed fillers. Presence of such charges within the structure of PANi lead to generation of electron delocalization and therefore high conductivity of PANi [50,51]. Additionally, peaks within ranges 1230–1239 cm^−1^ and 1287–1292 cm^−1^ are attributed to the C–N^+^ of polaronic structure and C–N stretch of the secondary aromatic amine of PANi, respectively, where these peaks showing higher intensity in the PANi- GO-ITN 25% and PANi- GO-ITN 50% structures that confirm presence of higher amount of C–N^+^ and C–N groups within the polymeric structure of reinforced PANi compared with neat PANi [23].

Furthermore, appeared peaks within regions 1453–1484 cm^−1^ and 1549–1566 cm^−1^ are correspond to the stretch of NH-benzenoid ring (B)-NH (C=C e C=N stretching of the benzene rings) and stretch of N=quinoid ring (Q)=N (C=C e C=N stretching of the quinoid ring) of PANi, respectively [52,53]. Higher intensity of these peaks in the FTIR spectrums of PANi- GO-ITN 25% and PANi- GO-ITN 50% compared with neat PANi showed that addition of GO-ITN to the PANi can significantly promote the overall features of PANi and improve the charge carrier of PANi’s polymeric structure which can lead to higher conductivity, and thus higher electromagnetic wave attenuation. Besides, peaks within the region between 1989–1994 cm^−1^ and 2090–2094 cm^−1^ are attributed to the C=C double bond carbon atoms and azide functional groups, respectively (i.e., $N^{-}\equiv N^{+}=N^{-}$) [23]. Also, peaks around 3229 cm^−1^ within the FTIR spectrums of PANi- GO-ITN 25% and PANi- GO-ITN 50% are attributed to hydroxyl functional groups (–OH) [54]. Obtained data from FTIR analysis confirmed the successful synthesis of PANi along with superior role of GO-ITN for enhancement of conductive parts of PANi’s structure.

Besides, XRD spectrum of PANi, PANi-GO-ITN 25%, and PANI- GO-ITN 50% can be seen in Figure 5b. As can be seen in this Figure, the diffraction pattern of PANi shows 2ϴ peaks at around 14.97, 20.59, and 25.18 that correspond to (112), (020), and (200) crystal planes of PANi, respectively. Presence of these peaks justify the successful fabrication of protonated emeraldine semi-crystalline structure of PANi [54,55,56]. More importantly, while PANi-GO-ITN 25% showed nearly same peaks compared with neat PANi, the XRD spectrum of modified PANi showed related peaks of the doped structure between Fe and W denoted as iron tungsten nitride. In this matter, peaks at 2ϴ of 27.91, 32.38, and 46.37 are attributed to the (222), (004), and (044) crystalline planes of iron tungsten nitride (i.e., W_96_Fe_40_N_8_) that is in-well accord with obtained data from FTIR, EDAX, and FESEM analyses and confirm the formation of an interconnected doped structure on the basal plane of GO. More details about this doped structure can be seen in Table S9.

**Figure 6:**
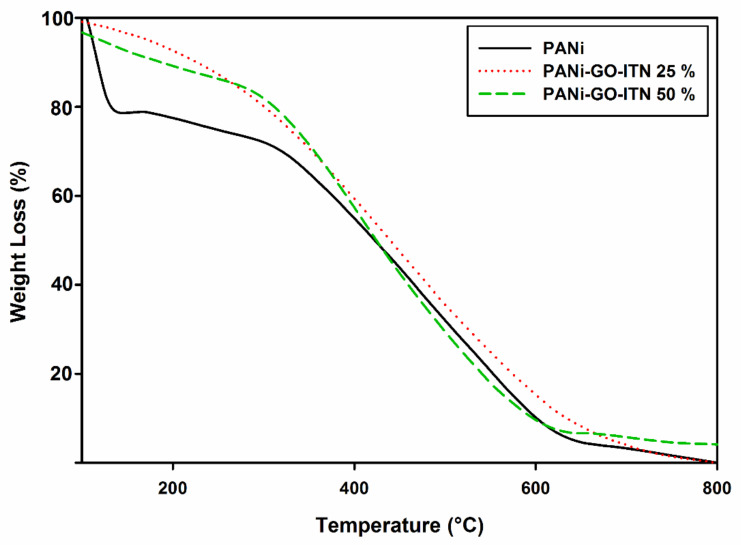
TGA analysis of PANi, PANi-GO-ITN 25%, and PANi-GO-ITN 50%.

Developed nanocomposites were examined via TGA analysis from ambient temperature up to 800 °C with rate 10 °C/min to evaluate the effect of GO-ITN addition on the thermal stability of PANi. As shown in Figure 6, neat PANi demonstrated three major weight losses. In this matter, the primary weight loss occurred at around 100 °C which is due to the loss of moisture. The second one occurred around 300 °C which is attributed to the evaporation of NMP from the nanocomposite. Additionally, the main weight loss occurred at around 580–600 °C that correspond to the decomposition of the polymeric structure to some side degradation products including methane, carbazole, acetylene, and N-phenylaniline [57,58]. Furthermore, upon addition of GO-ITN to the polymeric structure of PANi, the thermal stability of the final composition considerably improved, a view of these data can clearly be seen in Figure 6.

**Figure 7:**
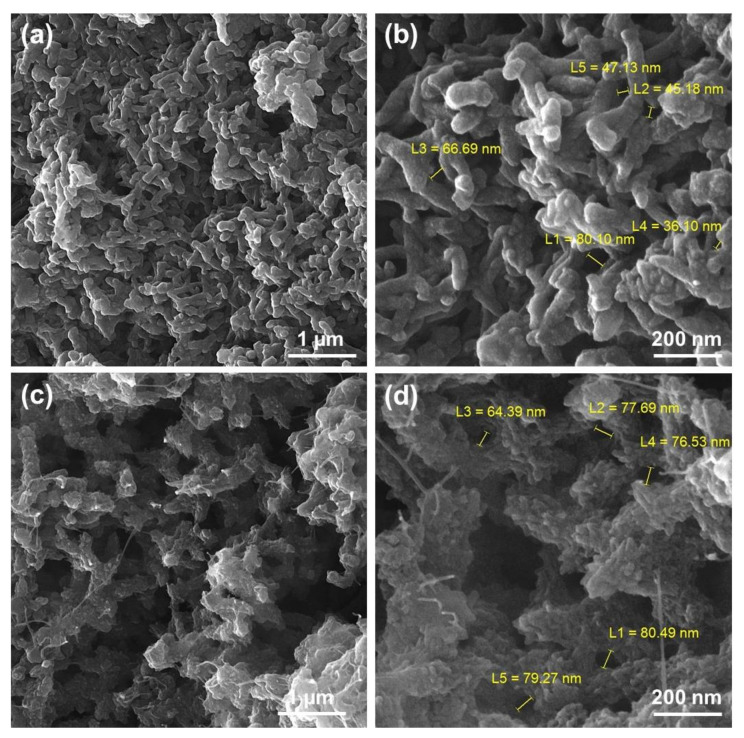
FESEM images of (**a**) and (**b**) PANi and (**c**) and (**d**) PANi-GO-ITN 50%.

In Figure 7, a view of FESEM images of PANi and modified PANi with GO-ITN hybrid flakes can be seen. As shown in Figure 6a,b, PANi is presenting a bean shaped structure with particle size less than 100 nm. On the other hand, reinforcement of PANi with GO-ITN significantly changes the morphology of PANi from nearly bean shaped tubes to planar nanostructure with a higher active surface area. This change in the morphology is due to the integration of GO-ITN hybrid flakes into the polymeric structure of PANi that can significantly improve the performance of developed nanostructures by improving their active surface areas and interaction with different media.

### 3.2. X-ray Shielding Effectiveness of Developed Shields

X-ray radiation is a harmful electromagnetic (EM) source that can adversely affect the human body and damage the DNA of living cells, while exposure to high X-ray dosage is highly carcinogenic. Additionally, widespread of radiotherapy and imaging technology via employing the X-ray radiation raised the concern about its harmful effects which require further attention for design and development of practical X-ray shields [9,15]. Herein, the performance of the developed shields against X-ray radiation at diverse tube voltages (i.e., 30, 40, and 60 kV) was examined to check their applicability against these kinds of harmful radiation sources, where specification of developed samples can be seen within the Table S1.

Attenuation of X-ray radiation occurs due to the absorption or deflection of photons from the irradiated beam. To design a practical shield, we should be aware of main factors that govern the attenuation process of X-ray radiation at diverse energy levels. Mainly, four factors governing the total attenuation of an X-ray beam, including nature of the radiation, density, atomic number, and electron per gram. In case of radiation’s nature, increase in the energy of the irradiated beams improve the total number of transmitted photons that can decrease the attenuation rate, while by increasing the density, atomic number, and electrons per gram of a shield, the number of passed photons will considerably decrease that lead to increase in the total attenuation rate of irradiated X-ray beams [59].

Besides, the absorption process of X-ray radiation occurs via photoelectric reaction at low energy levels (about 20 keV) or Compton scattering at high energy levels (above 100 keV) or even by mixture of both at moderate energy levels. At low energy levels of about 20 KeV, the photoelectric reaction is the predominates phenomena, regardless of the material atomic number. Further increase in the energy of the radiation decline photoelectric reactions within the absorber and improve the amount of Compton scattering which can eventually become the predominant phenomena at higher tube voltages; however, increase in the atomic number of the shield can improve the number of photoelectric reactions. The linear attenuation coefficient of an absorber is sum of coherent scattering, photoelectric reactions and Compton scattering (i.e., µ = µ_Coherent_ + µ_Photoelectric_ + µ_Compton_). What is more, when the photoelectric reaction is predominant, more irradiated beams will be absorbed compared with Compton scattering. The energy of the irradiated beams also has a linear relation with the attenuation rate, and by further increase in the energy of beams, the attenuation rate will decline accordingly [59].

Generally, increasing the radiation energy can improve the overall percentage of transmitted photons and decline the attenuation rate which is regardless of the type of interactions. However, materials with high atomic number will decrease the transmission by a further increase in the beam energy. An irradiated photon cannot eject an electron unless it has much more energy than the binding energy of the electron. Therefore, low energy photons will more likely transmitted compared with higher energy photons, where the weaker and stronger photons have less and more energy than the binding energy of the electrons, respectively [59].

The density of the absorber is the most crucial factor for attenuation of an incident X-ray beam. In fact, density determines the number of electrons that exist in specific thickness of the absorber (i.e., the higher the density, the higher the number of electrons), where the relation between density and attenuation is liner and further increase in the density will improve the attenuation rate accordingly. Compton reaction depends on the number of electrons within the material’s thickness. In this matter, a shield with more electrons (or higher density) absorb X-ray beams more impressively compared with material with least electrons [59].

**Figure 8:**
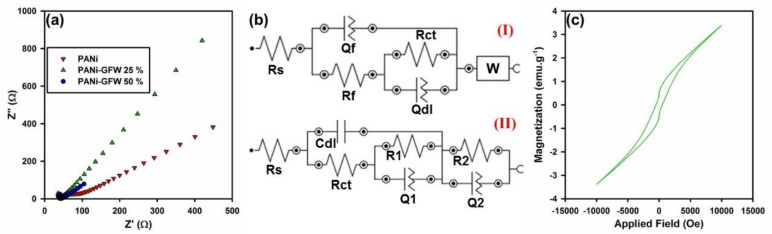
(**a**) Nyquist plots of PANi and reinforced PANi with diverse filler loadings of GO-ITN, (**b**) used circuits for measuring the electrical conductivity of (I) PANi and (II) reinforced PANi with 50 wt % GO-ITN, and (**c**) the outcome of VSM analysis for reinforced PANi with 50 wt % GO-ITN hybrid flakes.

Herein, we developed a hybrid material with ideal specifications that contains materials with high density electrical conductivity and atomic number where the complex hybrid structure also shows a bite of magnetic properties. The developed filler consists of decorated GO with hybrid iron tungsten nitride alloy that exhibit density of about 24.21 g·cm^−1^; this hybrid nanomaterial is an ideal absorber for attenuation of incident X-ray beams. Likewise, the electrical conductivity of developed specimens was evaluated using impedance spectroscopy (EIS), which follow up the changes of the electron transfer resistance during the fabrication process. For this purpose, the Nyquist plots (real part Z′ and imaginary part Z″) were obtained for prepared electrodes over a frequency range of 100 kHz to 0.1 Hz at an amplitude of 10 mV and an open circuit potential in the presence of PBS pH 7.0. A view of these data can be seen within the Figure 8a which shows that the magnitude of Z′ was reduced by further increase in the applied frequency range. On the other hand, the impedance spectrum in the Nyquist plot elucidated a semicircle region at higher frequencies related to limited process of electron transfer and a linear part at lower frequency range corresponding to a diffusion-limited process. Semicircle diameter (R_ct_) is an indication for the electron-transfer resistance, which is the main criterion for measurement of electrical conductivity. Thus, the electrical conductivity of a material can be calculated via the formula [60]

σ = L/(R·A)(2)

where L and A are the thickness and geometric surface area of the sample, respectively; σ is the electrical conductivity (S·cm^−1^), and R is the resistance (Ω) of the substrate. The EIS experimental data are well-fitted into appropriate equivalent circuit models (Figure 8b), where the circuit (I) and (II) were considered for calculating the electrical conductivity of PANi and modified PANi with GO-ITN hybrid flakes, respectively. The estimated data are analyzed, and the related parameters such as R_s_, R_ct_, and C_dl_ are tabulated within Table 1. The small semicircles in a higher frequency range indicate a negligible charge transfer resistance in electrode systems which highlighted the potential conducting behavior of PANi. These data showed that modification of PANi with 25 and 50 wt % GO-ITN can increase the electrical conductivity of the final composition about 0.65 and 5.77 S·cm^−1^ which exhibit about 14.840% and 131.73% increase in the electrical conductivity compared with neat PANi, respectively. These outcomes highlight the improvement in the electron per gram of the material and presence of higher charge carriers on the surface of the absorber that is ideal for improving the X-ray absorption rate of the final composition.

In addition, the outcome of VSM analysis for the reinforced PANi with 50 wt % GO-ITN hybrid flakes can be seen in Figure 8c. As can be seen in this Figure, the reinforced composite of PANi shows saturation magnetization (M_s_), remanence (M_r_), coercivity (H_c_), switching field distribution (SFD) (SFD = ∆H/H_c_), and squareness (M_r_/M_s_) of about 3.4 emu·g^−1^, 0.653 emu·g^−1^, 490 Oe, 2.142 and 0.192, respectively. Additionally, these data showed that the developed platform exhibiting high electrical conductivity and appropriate magnetic permeability that can improve the uptake or attenuation of EM waves when being coupled with a high density of the main X-ray absorbing component (i.e., iron tungsten nitride) that has density of about 24.21 g·cm^−3^.

**Figure 9:**
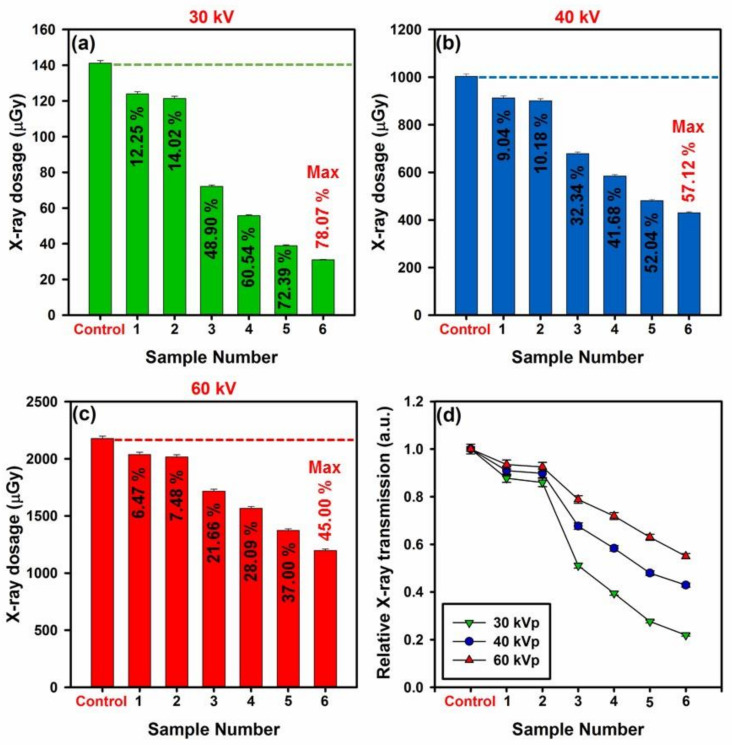
Overall rate of X-ray attenuation via the developed specimens at (**a**) 30 kVp, (**b**) 40 kVp, (**c**) 60 kVp X-ray tube voltages along with (**d**) relative X-ray transmission at diverse tube voltages.

In Figure 9, the overall X-ray attenuation rate of developed shields can be seen. In this matter, the fabricated PANi-based shields were exposed to X-ray radiation with diverse dosage, and the overall rate of X-ray attenuation for each sample was recorded by comparing with the control value (detected X-ray beams by the sensor without any shield). In Figure 9a–c, the overall rate of X-ray attenuation for developed samples at 30, 40, and 60 kV can be seen, respectively. As can be seen in this figure, by further increase in the filler loading of GO-ITN and thickness of samples, the overall rate of X-ray attenuation was considerably increased. In this regard, at 30 kV, sample 4 and 6 absorbed about 85.49 and 110.24 µGy of the incident X-ray waves (compared with control 141.2 µGy) that correspond to 60.545% and 78.073% absorption of the total irradiated X-ray waves. On the other hand, by further increase in the dosage of irradiated X-ray waves, the overall absorption rate has declined, where at 40 kV/60 kV, sample 4 and 6 absorbed about 41.684%/28.099% and 57.128%/44.995% of the incident X-ray waves, respectively. In Figure 9d, the relative X-ray transmission of the samples can be seen. As shown, increase in the filler loading which correspond to increase in respective density and total number of electrons per thickness of the shield led to decrease in the total X-ray transmission. These outcomes highlighting the potential of the developed shields for absorption of X-ray radiation even at low thicknesses, about ~1 mm, which is vital for reaching the standard for fabrication of lightweight and efficient absorbers; more details about X-ray absorption rate of diverse samples can be seen within the Table S10.

### 3.3. Anti-Bacterial, Anti-Fungal, and Toxicity Evaluations of Developed Shields

**Figure 10:**
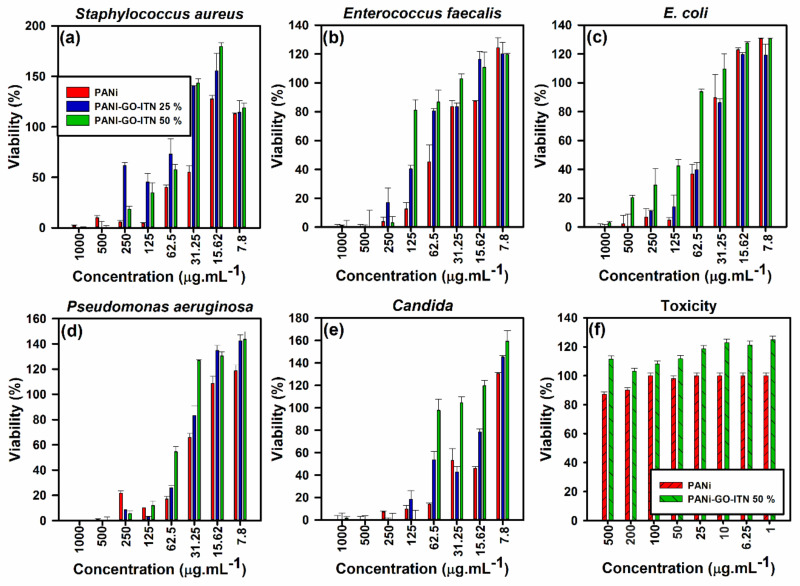
Performance of the developed shields against (**a**) *Staphylococcus aureus*, (**b**) *Enterococcus faecalis*, (**c**) *E. coli*, (**d**) *Psedomonas aeruginosa*, and (**e**) *Candida* along with (**f**) toxicity evaluation of PANi and reinforced PANi with 50 wt % GO-ITN.

Recently developed X-ray shields have a tremendous weak point which is their superior toxicity that can significantly affect the health of consumers. Moreover, nosocomial infections can also affect doctors and nurses all of who interfacing with diverse kinds of bacteria and yeast, therefore, it is vital to develop non-toxic shields that protect the consumer against bacteria and yeast cultures. In this section, the anti-bacterial, anti-fungal (by mean of MIC, MBC, and MFC) and toxicity of the developed shields were examined and their performance against selected gram-positive bacteria (i.e., *Staphyloccus aureus* and *Enterococcus faecalis*), gram negative bacteria (i.e., *E. coli* and *Pseudomonas aeruginosa*), and yeast (i.e., *Candida*) were examined, where a view of these results can be seen within the Figure 10 and Table 2. As can be seen in Figure 10 and Table 2, all of the developed shields showed high potential for the removal of selected bacteria and yeast at concentration of 1000 µg·mL^−1^, while potential of the developed shields was variated for diverse microorganism depend on their type and the concertation of the anti-bacterial/anti-fungal agent.

In case of gram positive bacteria, PANi, PANi-GO-ITN 25%, and PANi- GO-ITN 50% showed MIC/MBC at concentrations of about 125/125, 500/500, and 500/500 µg·mL^−1^ for *Staphylococcus aureus* (Figure 10a), respectively, and 250/250, 500/500, and 250/250 µg·mL^−1^ for *Enterococcus faecalis* (Figure 10b), respectively. In this case, all of developed shields showed bactericide behavior against gram positive bacteria, while PANi showed better anti-bacterial performance against the selected gram positive bacteria compared with reinforced ones, which could be due to the higher degree of protonation owing to the integration of hybrid GO-ITN flakes with polymeric structure of PANi that can increase the electrical conductivity and overall amount of positive charges on the surface of modified PANi. This fact was proved via increase in the intensity of FTIR peaks related to N=quinoid ring (Q)=N/B–N^+^H-B bonds (1108–1125 cm^−1^), C–N^+^ (1230–1239 cm^−1^), NH-benzenoid ring (B)–NH (1453–1484 cm^−1^), and N=quinoid ring (Q)=N (1549–1566 cm^−1^) along with electrical conductivity of modified PANi compared with neat PANi due to the addition of GO-ITN flakes.

On the other hand, developed platforms showed a bite better performance against gram-negative bacteria compared with gram-positive ones, which are due to the higher degree of positive charges on their surfaces [23]. In this regard, PANi, PANi- GO-ITN 25%, and PANi- GO-ITN 50% showed MIC/MBC at concentrations of 125/125, 500/500, and 1000/1000 µg·mL^−1^ for *E. coli* (Figure 10c), respectively, and 500/500, 125/125, and 250/250 µg·mL^−1^ for *Pseudomonas aeruginosa* (Figure 10d), respectively. In fact, developed shields showed bactericide behavior against gram negative bacteria, while they showed higher bacterial inhabitation rate against *Pseudomonas aeruginosa* compared with *E. coli*. What is more, the developed platforms showed perfect effectiveness against the selected yeast (i.e., *Candida*) where PANi, PANi- GO-ITN 25% and PANi- GO-ITN 50% showed MIC/MFC at concentrations of 125/125, 250/250, and 125/125 µg·mL^−1^, respectively (Figure 10e).

These outcomes showed the superior performance of the developed shields for simultaneous absorption of X-ray irradiation and removal of microorganisms from their surfaces which is vital for development of practical radiation shields for hospital, radiotherapy, and/or radiology applications. More importantly, reinforced shields showed entirely non-toxic behavior in interaction with selected living cells, and even they improved the viability of cells, while PANi showed toxicity against them at diverse concentrations (Figure 10f). This fact could be one of the reasons for better anti-bacterial inhabitation effectiveness of neat PANi compared with reinforced PANi that showing less toxicity.

## 4. Conclusions

X-ray radiation can significantly deteriorate living cells by damaging their DNA and lead to formation of cancer in diverse parts of the body, thus, its barebone essentials to protect vulnerable sources from them through practical strategies. Herein, PANi was reinforced with diverse filler loadings (i.e., 25 and 50 wt %) of dense hybrid iron tungsten nitride flakes toward development of non-toxic, anti-bacterial, and anti-fungal shield that can attenuate most of irradiated X-ray beams. In this matter, the successful fabrication of developed materials was confirmed via diverse characterization techniques, and thence specimens were subjected to different evaluations. The outcome of primary analyses showed that samples containing 25 and 50 wt % GO-ITN showing 14.84% and 131.73% increase in the electrical conductivity along with weak magnetic properties which is vital for high yield absorption of electromagnetic (EM) waves. Likewise, achieved results from X-ray attenuation examination showed that 1.2 mm thick samples containing 25 and 50 wt % GO-ITN showing 60.454%/41.684%/28.099% and 78.073%/57.128%/44.995% decrease in the total amplitude of the irradiated beams at 30/40/60 kV tube voltage compared with control, respectively. More importantly, developed shields completely removed selected microorganism at concentration of 1000 µg·mL^−1^ and showed entirely non-toxic nature against the selected living cells. Such non-toxic and highly efficient X-ray shield can simultaneously block X-ray beams and control nosocomial infections which meet the urgent worldwide requirement for multi-functional X-ray absorbing shields.

## Figures and Tables

**Table 1:** Electrochemical parameters extracted from fitting the EIS data on modified electrodes.

Electrode	R_s_ (Ω)	R_ct_ (Ω)	C_dl_ (μF)	σ (S·cm^−1^)
PANi	642 ± 2	45.6 ± 2	-	4.38
PANi-GFW 25%	313 ± 1	39.7 ± 0.1	2.88 ± 0.05	5.03
PANi-GFW 50%	113 ± 1	1.97 ± 0.1	11.7 ± 0.05	10.15

**Table 2:** MIC and MBC/MFC values of the developed shields against selected microorganisms.

Microorganism		PANi (µg/mL)			PANi-GFW 25% (µg/mL)			PANi-GFW 50% (µg/mL)		
		MIC	MBC	MFC	MIC	MBC	MFC	MIC	MBC	MFC
Gram positive	*Staphyloccus aureus*	125	>125	-	500	500	-	500	500	-
	*Enterococcus faecalis*	250	>250	-	500	500	-	250	250	-
Gram negative	*E. coli*	125	>125	-	500	500	-	1000	1000	-
	*Pseudomonas aeruginosa*	500	>500	-	125	125	-	250	250	-
Yeast	*Candida*	125	-	>125	250	-	>250	125	-	>125

## Supplementary Materials

The following are available online at https://www.mdpi.com/2073-4360/12/6/1407/s1, Figure S1. Formation of GO out of pure graphite flakes, Table S1. Specification of developed samples, Table S2. I_D_/I_G_ ratio obtained by current method compared with literature, Table S3. Element analysis of fabricated GO via XPS, Table S4. De-convolution of peak C1s into five diverse segments, Table S5. De-convolution of peak N1s into three diverse segments, Table S6. De-convolution of peak O1s into four diverse segments, Table S7. XPS and XRD results comparison between previous methods and current study, Table S8. EDAX analysis of hybrid GO-ITN flakes, Table S9. Specification of appeared peaks of iron tungsten nitride’s structure, Table S10. Performance of the developed shields against incident X-ray waves.
