# Supplementary material for: Superior X-ray Radiation Shielding Effectiveness of Biocompatible Polyaniline Reinforced with Hybrid Graphene Oxide-Iron Tungsten Nitride Flakes
Source: Polymers (Basel). 2020 Jun 23;12(6):1407. doi: 10.3390/polym12061407 (PMC7361692; doi:10.3390/polym12061407)
Supplement: Supplementary file 1 [file polymers-12-01407-s001.pdf]

Supporting Information

Superior X-ray Radiation Shielding Effectiveness of Biocompatible Polyaniline Reinforced with Hybrid Graphene Oxide-Iron Tungsten Nitride Flakes

Seyyed Alireza Hashemi [1, 2, *], Seyyed Mojtaba Mousavi [3, 2], Reza Faghihi [4, 5], Mohammad Arjmand [6], Mansour Rahsepar [7], Sonia Bahrani [2], Seeram Ramakrishna [1], and Chin Wei Lai [8]

[1] Department of Mechanical Engineering, Center for Nanofibers and Nanotechnology, National University of Singapore, Singapore; seeram@nus.edu.sg

[2] Department of Medical Nanotechnology, School of Advanced Medical Sciences and Technologies, Shiraz University of Medical Sciences, Shiraz, Iran; kmepo.smm@gmail.com (S.M.M.); s.bahrani22@gmail.com (S.B.)

[3] Department of Chemical Engineering, National Taiwan University of Science and Technology, Taiwan

[4] Nuclear Engineering Department, Shiraz University, Shiraz, Iran. 71936-16548; faghihir@shirazu.ac.ir

[5] Radiation Research Center, Shiraz University, Shiraz, Iran. 71936-16548

[6] School of Engineering, University of British Columbia, Kelowna, BC, V1V 1V7, Canada; mohammad.arjmand@ubc.ca

[7] Department of Materials Science and Engineering, School of Engineering, Shiraz University, Zand Boulevard, Shiraz, Iran. 71348-51154; mansour.rahsepar@gmail.com

[8] Nanotechnology & Catalysis Research Center, University of Malaya, Malaysia; cwlai@um.edu.my

* Correspondence: sa_hashemi@sums.ac.ir (S.A.H.); Tel: 0098 9398432509

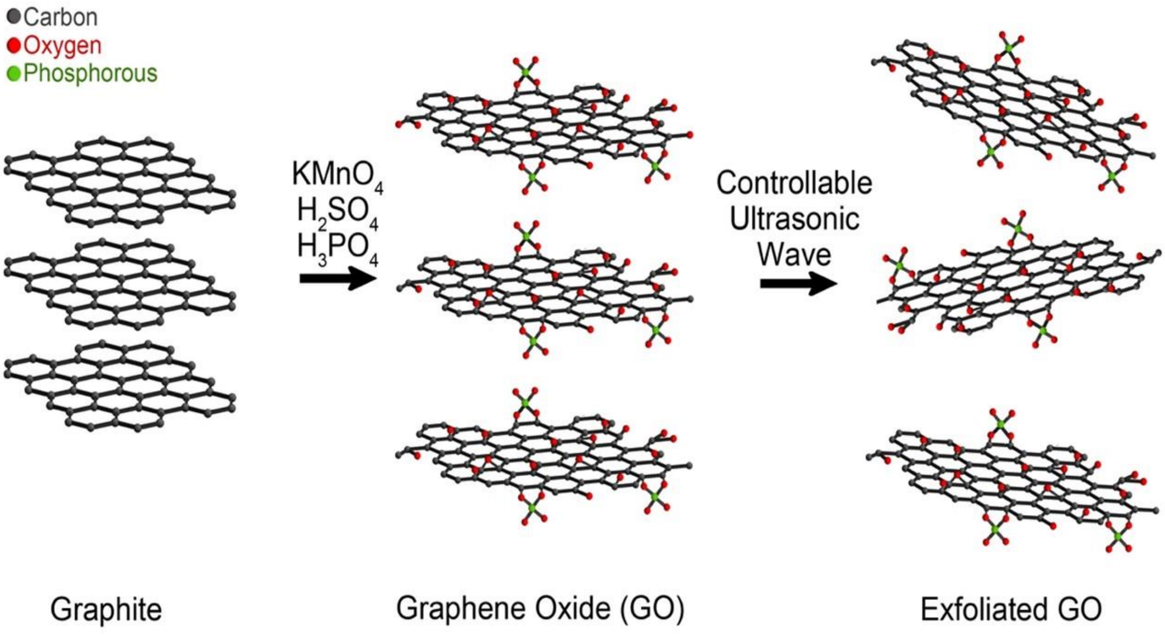

Figure S1. Formation of GO out of pure graphite flakes.

Table S1. Specification of developed samples.

Sample Number	GO-ITN (wt%)	Thickness (mm)	Diameter (cm)
1	0	0.95	2
2	0	1.2	2
3	25	0.95	2
4	25	1.2	2
5	50	0.95	2
6	50	1.2	2

Table S2. I_D/I_G ratio obtained by current method compared with literature.

Production method	Type of Graphene	I_D/I_G	Ref.
Hummers	rGO	0.88 - 0.92	[1]
Hummers	GO	1.14 – 1.19	[2]
Hummers	rGO	1.28 – 2.70	[2]
Modified Hummers	GO	1.42 - 1.88	[3]
Modified Hummers	rGO	1.12 - 1.24	[3]
Modified Hummers	GO	0.97	[4]
Modified Hummers	rGO	1.15 – 1.4	[4]
Modified Hummers	GO	1.07	[5]
Current study	GO	0.835	-

Table S3. Element analysis of fabricated GO via XPS.

Name	Peak BE	FWHM eV	Area (p) CPS.eV	Atomic %
C1s	284.72	1.65	57958.50	68.36
O1s	532.65	2.07	63486.40	31.00
N1s	401.05	0.94	850.10	0.65

Table S4. De-convolution of peak C1s into five diverse segments.

Peak / eV	FWHM eV	Area (p) CPS.eV	Atomic %
Sp^1 (283.78 eV)	1.65	1902.42	3.33
Sp^2 (284.78 eV)	1.48	23762.61	41.62
Sp^3 (286.85 eV)	1.35	21682.91	38.03
C=O (288.61 eV)	1.74	8006.78	14.06
O-C=O (290.48 eV)	2.17	1682.09	2.96

Table S5. De-convolution of peak N1s into three diverse segments.

Peak / eV	FWHM eV	Area (p) CPS.eV	Atomic %
Pyridinic Nitrogen (397.33 eV)	1.92	127.18	19.87
Pyrrolic Nitrogen (400.19 eV)	1.92	260.53	40.79
Pryidine-N-Oxide (402.08 eV)	1.92	251.00	39.35

Table S6. De-convolution of peak O1s into four diverse segments.

Peak / eV	FWHM eV	Area (p) CPS.eV	Atomic %
C=O (531.47 eV)	1.54	11234.61	18.08
C-OH (532.66 eV)	1.54	39625.13	63.83

C-O (533.74 eV)	1.54	8335.48	13.44
C-OH (535.53 eV)	1.92	2877.73	4.65

Table S7. XPS and XRD results comparison between previous methods and current study.

**Method**	Type of graphene	sp^1 hybridization (%)	sp^2 hybridization (%)	sp^3 hybridization (%)	d-spacing (Å)	Ref.
Hummer	GO	-	39	61	8	[6]
Hummer	rGO	14.6	67.8	17.6	8.133	[7]
Modified Hummer	GO	-	37	63	9	[6]
Modified Hummer	GO	35	-	65	9.06	[8]
Improved Method	GO	-	31	69	9.5	[6]
Hofmann	rGO	14.2	67.1	18.7	7.226	[7]
Staudenmaier	rGO	13.2	69.3	17.5	7.084	[7]
Current study	GO	3.3	41.6	55.1	3.8499	-

Table S8. EDAX analysis of hybrid GO-ITN flakes.

**Element**	**Intensity**	**Weight %**	**Atomic %**
C	17.9	8.02	31.26
N	3.1	1.87	6.26
O	64.8	11.87	34.74
Fe	352.3	13.39	11.23
W	247.5	64.85	16.52

Table S9. Specification of appeared peaks of iron tungsten nitride's structure.

Position (2θ)	d-spacing (Å)	(h k l)	Chemical formula	Crystalline size (Å)	Micro strain (%)
27.9181	3.19588	(2 2 2)	$W_{96}Fe_{40}N_8$	157.2079	1.01645
32.3874	2.76435	(0 0 4)	$W_{96}Fe_{40}N_8$	392.3921	0.352243
46.3768	1.95791	(0 4 4)	$W_{96}Fe_{40}N_8$	337.9722	0.289655

Peaks were extracted from refence number 96-200-6776 related to the iron tungsten nitride (i.e., $W_{96}Fe_{40}N_8$) that exhibit cubic structure and its crystalline specifications are as follow: a (Å): 11.11, b (Å): 11.11, (Å): 11.11, alpha (°): 90, beta (°): 90, gamma (°): 90, calculated density: 24.21 g.cm^{-3} and volume of cells 1371.33 × 10^6 pm^3.

Table S10. Performance of the developed shields against incident X-ray waves.

**Sample**	**Detected X-ray (µGy)**			**Attenuated X-ray (µGy)**			**X-ray Absorption (%)**		
	**30 kV**	**40 kV**	**60kV**	**30 kV**	**40 kV**	**60kV**	**30 kV**	**40 kV**	**60kV**
**Control**	141.2	1003	2178	-	-	-	-	-	-
**1**	123.9	912.3	2037	17.3	90.7	141	12.252	9.0428	6.473
**2**	121.4	900.8	2015	19.8	102.2	163	14.022	10.189	7.483
**3**	72.14	678.6	1717	69.06	324.4	461	48.909	32.342	21.166
**4**	55.71	584.9	1566	85.49	418.1	612	60.545	41.684	28.099
**5**	38.98	481.01	1372	102.22	521.99	806	72.393	52.042	37.006
**6**	30.96	430	1198	110.24	573	980	78.073	57.128	44.995

References:

1. Gupta, B.; Kumar, N.; Panda, K.; Kanan, V.; Joshi, S.; Visoly-Fisher, I. Role of oxygen functional groups in reduced graphene oxide for lubrication. *Scientific Reports* **2017**, *7*, 45030.
2. Eigler, S.; Dotzer, C.; Hirsch, A. Visualization of defect densities in reduced graphene oxide. *Carbon* **2012**, *50*, 3666-3673.
3. King, A.A.; Davies, B.R.; Noorbehesht, N.; Newman, P.; Church, T.L.; Harris, A.T.; Razal, J.M.; Minett, A.I. A New Raman Metric for the Characterisation of Graphene oxide and its Derivatives. *Scientific reports* **2016**, *6*.
4. Rajagopalan, B.; Chung, J.S. Reduced chemically modified graphene oxide for supercapacitor electrode. *Nanoscale research letters* **2014**, *9*, 535.
5. Gupta, V.; Sharma, N.; Singh, U.; Arif, M.; Singh, A. Synthesis and characterization of Graphene Oxide. *Optik-International Journal for Light and Electron Optics* **2017**.
6. Marcano, D.C.; Kosynkin, D.V.; Berlin, J.M.; Sinitskii, A.; Sun, Z.; Slesarev, A.; Alemany, L.B.; Lu, W.; Tour, J.M. Improved synthesis of graphene oxide. **2010**.
7. Poh, H.L.; Šaněk, F.; Ambrosi, A.; Zhao, G.; Sofer, Z.; Pumera, M. Graphenes prepared by Staudenmaier, Hofmann and Hummers methods with consequent thermal exfoliation exhibit very different electrochemical properties. *Nanoscale* **2012**, *4*, 3515-3522.
8. Tao, C.-a.; Wang, J.; Qin, S.; Lv, Y.; Long, Y.; Zhu, H.; Jiang, Z. Fabrication of pH-sensitive graphene oxide–drug supramolecular hydrogels as controlled release systems. *Journal of Materials Chemistry* **2012**, *22*, 24856-24861.
